# Clinicopathological and immunological characterization of RNA m^6^A methylation regulators in ovarian cancer

**DOI:** 10.1002/mgg3.1547

**Published:** 2020-11-22

**Authors:** Qingying Wang, Qinyi Zhang, Qingxian Li, Jing Zhang, Jiawen Zhang

**Affiliations:** ^1^ Department of Obstetrics and Gynecology Shanghai Tenth People’s Hospital School of Medicine Tongji University Shanghai China; ^2^ Department of Obstetrics and Gynecology Shanghai General Hospital Shanghai Jiao Tong University Shanghai China; ^3^ Department of Gynecology and Obstetrics Putuo Hospital Shanghai University of Traditional Chinese Medicine Shanghai China; ^4^ Department of Integrated Therapy Shanghai Cancer Center Fudan University Shanghai China; ^5^ Department of Oncology Shanghai Medical College Fudan University Shanghai China

**Keywords:** immune infiltration, m^6^A regulators, ovarian cancer, survival

## Abstract

**Background:**

N^6^‐methyladenosine (m^6^A) modification is one of the critical gene regulatory mechanisms implicated in cancer biology. However, the roles of m^6^A regulators in ovarian cancer are still poorly understood.

**Methods:**

We integrated multiple databases including Gene Expression Omnibus (GEO), ROC Plotter, Kaplan‐Meier Plotter, and Tumor Immune Estimation Resource (TIMER) to explore clinicopathological significance of m^6^A regulators in ovarian cancer.

**Results:**

We showed that alterations in the expression of m^6^A regulators were related to the malignancy and poor prognosis of ovarian cancer. We found decreased YTHDC1 and increased RBM15 expressions were associated with ovarian cancer cell metastases and HNRNPC was a predictor of paclitaxel resistance. Moreover, dysregulated m^6^A regulators were enriched in the activation of cancer‐related pathways. Our results further demonstrated that the level of immune cell infiltration and the expression of various immune gene markers were closely associated with the expressions of specific m^6^A regulators (RBM15B, ZC3H13, YTHDF1, and IGF2BP1).

**Conclusions:**

Our study establishes a new prognostic profile of ovarian cancer patients based on m^6^A regulators, and highlights the potential roles of m^6^A regulators in ovarian cancer development.

## Graphical abstract

m^6^A regulators play important roles in ovarian cancer cell biology.

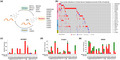

## 1 INTRODUCTION

Ovarian cancer is the first leading cause for death of gynecological cancers worldwide, with an estimated 295,000 new cases and 185,000 deaths in 2018 (Bray et al., 2018). Although the therapy modalities have been greatly improved, more than 70% of patients with advanced stages still have tumor recurrence, and the 5‐year overall survival rate of ovarian cancer patients is still very low (Lheureux et al., 2019). Tumor‐related immune modulation plays an important role in ovarian cancer. Tumor‐infiltrating lymphocytes (TILs), including CD8^+^ T cells, macrophages, neutrophils, and dendritic cells affect the prognosis and efficacy of immunochemotherapy (Santoiemma & Powell, 2015). Therefore, it is an urgent need to find new biomarkers and immune‐related targets for the prognosis and treatment of ovarian cancer.

N^6^‐methyladenosine (m^6^A) is the most common post‐transcriptional modification in mRNA. It affects RNA metabolism, such as alternative splicing, translation, and degradation (Roundtree et al., 2017). The modification of m^6^A is catalyzed by different types of regulators, including m^6^A methyltransferases (METTL3/14, RBM15/15B, VIRMA, WTAP, and ZC3H13, termed as ‘writers’), demethylases (FTO and ALKBH5, termed as ‘erasers’), and RNA binding proteins (HNRNPA2B1, HNRNPC, IGF2BP1/2/3, YTHDC1/2, YTHDF1/2/3, and RBMX, termed as ‘readers’) (Meyer & Jaffrey, 2017; Zaccara et al., 2019). The dynamic modification in m^6^A mediated by these regulators not only plays important roles in the development of oocytes and cerebellum but also plays essential roles in regulating cell proliferation and migration, leading to the malignant progression of various cancers and treatment resistance (Chen et al., 2019; Lan et al., 2019). The latest studies also revealed the connection between m^6^A regulators and tumor immune‐cell infiltration (Han et al., 2019; Li et al., 2020; Wang et al., 2019, 2020; Winkler et al., 2019). For example, inhibition of METTL3/14 promoted IFN‐γ‐STAT1‐IRF1 signaling and enhanced response to anti‐PD‐1 treatment in colorectal cancer (Wang et al., 2020). ALKBH5 inhibitor could heighten the efficacy of cancer immunotherapy (Li et al., 2020). In recent years, comprehensive analysis of the clinical relevance and molecular characteristics of m^6^A regulators across several cancer types has been reported (Chai et al., 2019; Kwok et al., 2017; Li et al., 2019; Su et al., 2019; Zhou et al., 2019). However, their roles in ovarian tumorigenesis remain unclear.

Here, we systematically assessed the expression pattern, clinicopathological, and prognostic relevance of m^6^A regulators through extensive bioinformatics analyses. We revealed the predictive value and clinical significance of m^6^A regulators in ovarian cancer. Importantly, our results also indicated that the level of immune cell infiltration and the expression of various immune gene markers were closely related to the expression of specific m^6^A regulators.

## 2 MATERIALS AND METHODS

### 2.1 Data acquisition

The TCGA‐OV dataset used in our study were downloaded from The Cancer Genome Atlas (TCGA) data portal (https://cancergenome.nih.gov/). Genetic data were obtained from cBioPortal (https://www.cbioportal.org/) (Cerami et al., 2012; Gao et al., 2013). Nine sets of microarrays (GSE14407, GSE12470, GSE69428, GSE84829, GSE28979, GSE9891, GSE73168, GSE30587, and GSE51373) were extracted from the Gene Expression Omnibus (GEO) datasets (http://www.ncbi.nlm.nih.gov/geo/).

### 2.2 Selection of RNA m^6^A methylation regulators

We collated a list of 20 m^6^A regulators from recently published literature, including 11 readers, 7 writers, and 2 erasers (Yang et al., 2018). We extracted the available mRNA expression data in GEO datasets of these genes and the clinicopathological information of the samples for subsequent bioinformatics analysis.

### 2.3 Bioinformatic analysis of expression profiles

Genetic status data available at TCGA database were assessed using the cBioPortal to investigate the genomic profiling of m^6^A regulators in ovarian cancer. The GEO datasets were used to evaluate the expression alterations of m^6^A regulators in normal and tumor tissues. GSE14407 evaluated the differential gene expression between 12 laser capture microdissected serous ovarian cancers and 12 ovarian surface epithelial cells. GSE12470 evaluated the differential gene expression between 43 serous ovarian cancer and 10 normal peritoneum samples. GSE69428 compared gene expression profiles of high‐grade serous ovarian cancer (HGSOC) and paired normal oviduct samples from 10 independent patients. GSE84829 assessed gene expression patterns in 3 ascitic fluid‐isolated mesothelial cell samples obtained from stage III/IV ovarian serous carcinoma patients and 3 control peritoneal mesothelial cell samples isolated from omentum obtained from non‐oncologic patients. GSE28979 assessed gene expression patterns in 3 normal mouse fallopian tube oviduct and 3 early tumors from fallopian tubes of Dicer/PTEN knockout mice. GSE9891 analyzed the correlation between pathological grades/stages and expression level of m^6^A regulators in 285 ovarian cancer samples. GSE73168 evaluated the differential gene expression between 12 HGSOC primary tumor cells and 12 HGSOC ascites tumor cells. GSE30587 assessed gene expression patterns in 9 matched pairs of primary ovarian tumors and metastases from the omentum. GSE51373 evaluated the differential gene expression between 12 chemotherapy‐resistant and 16 chemotherapy‐sensitive HGSOC samples.

### 2.4 Receiver operating characteristics (ROC) Plotter

The ROC Plotter online platform (http://www.rocplot.org/) was used to identify specific m^6^A regulators which predicts benefit from chemotherapy (Fekete & Győrffy, 2019). The platform integrates multiple gene expression datasets at transcriptome level and contains 2369 ovarian cancer patients with treatment and response data.

### 2.5 Kaplan‐Meier Plotter analysis

Kaplan‐Meier plotter database (http://kmplot.com/analysis/) was used to investigate the prognostic value of m^6^A regulators in patients with ovarian cancer (Nagy et al., 2018). The hazard ratio (HR) with 95% confidence intervals (CI) and log‐rank *p*‐value were estimated.

### 2.6 TIMER database analysis

The TIMER online tool (https://cistrome.shinyapps.io/timer/) is a comprehensive resource for systematic analysis of immune infiltrates and contains 10,897 samples across 32 cancer types from TCGA (Li et al., 2016; Li, Fan, et al., 2017). It was used to analyze the correlation of m^6^A regulators with the abundance of immune cell infiltrates, including B cells, CD4^+^ T cells, CD8^+^ T cells, neutrophils, macrophages, and dendritic cells. Additionally, correlations between the expression of m^6^A regulators and various immune gene markers were explored via correlation modules. The gene expression level was displayed with log2 RSEM.

### 2.7 Gene set enrichment analysis

The biological functions potentially regulated by m^6^A regulators in ovarian cancer were evaluated by GSEA v3.0 software (Mootha et al., 2003; Subramanian et al., 2005). Hallmark gene sets and KEGG gene sets deposited in the GSEA Molecular Signatures Database v7.0 (MSigDB) were used.

### 2.8 Statistical analysis

One‐way ANOVA was used to compare the expression level of normal and tumor samples in GEO dataset. Student paired t test was used to compare the expression level in ovarian cancer for grade and stage. Chi‐square tests were used to compare the distribution of grade and stage between high‐ and low‐expression level groups. The expression of m^6^A regulators and therapy response were compared using ROC and Mann–Whitney tests. Survival rates were assessed using Kaplan–Meier curves and the log‐rank test. The correlation of m^6^A regulators with immune infiltration level and various immune gene markers was determined by Spearman's correlation. The data were analyzed using GraphPad Prism version 6.01 (GraphPad Software, Inc.) and presented as mean ± SD. *p*‐values <0.05 were considered statistically significant.

## 3 RESULTS

### 3.1 Expression profiles and clinical relevance of m^6^A regulators in ovarian cancer

**FIGURE 1:**
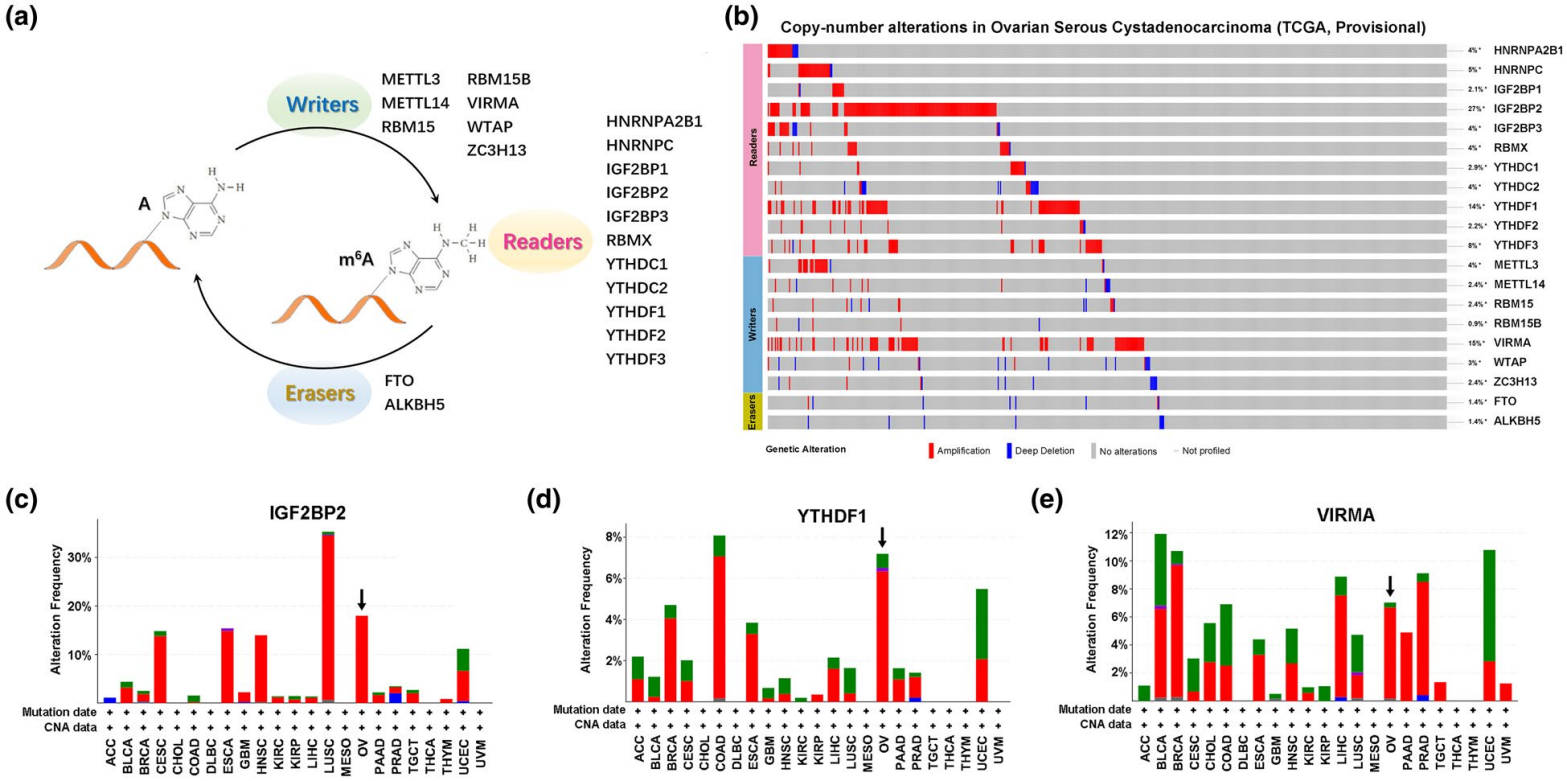
Genetic profiles of m^6^A regulators in ovarian cancer. (a) Diagram of m^6^A regulators analyzed in current study. (b) Genetic alterations of m^6^A regulators in ovarian cancer available at TCGA database by using cBioPortal (http://cbioportal.org). (c–e) Genetic alterations of IGF2BP2 (c), YTHDF1 (d), and VIRMA (e) across 23 cancer types. ACC, adrenocortical carcinoma; BLCA, bladder urothelial carcinoma; BRCA, breast invasive carcinoma; CESC, cervical squamous cell carcinoma and endocervical adenocarcinoma; CHOL, cholangio carcinoma; COAD, colon adenocarcinoma; DLBC, diffuse large B‐cell lymphoma; ESCA, esophageal carcinoma; GBM, glioblastoma multiforme; HNSC, head and neck squamous cell carcinoma; KIRC, kidney renal clear cell carcinoma; KIRP, kidney renal papillary cell carcinoma; LIHC, liver hepatocellular carcinoma; LUSC, lung squamous cell carcinoma; MESO, mesothelioma; OV, ovarian serous cystadenocarcinoma; PAAD, pancreatic adenocarcinoma; PRAD, prostate adenocarcinoma; TGCT, testicular germ cell tumors; THCA, thyroid carcinoma; THYM, thymoma; UCEC, uterine corpus endometrial carcinoma; UVM, uveal melanoma

In light of the crucial biological functions of m^6^A regulators in tumorigenesis, we systematically explored the genetic status and expression profile of each individual m^6^A regulator in ovarian cancer. We selected 20 well‐characterized m^6^A regulatory genes for analysis in current study, including 11 readers, 7 writers, and 2 erasers (Figure 1a). The genetic alteration of m^6^A regulators was first determined in the ovarian cancer patient cohort from TCGA database using cBioPortal. We found that IGF2BP2, YTHDF1, and VIRMA showed higher percentage of amplification, whereas the other m^6^A regulators had the lower frequency of overall mutation, ranging from 0.9 to 8.0% (Figure 1b‐e). In contrary to the relatively rare genetic mutations, more than half of m^6^A regulators showed significant alterations in mRNA expression level between normal and cancer samples.

**FIGURE 2:**
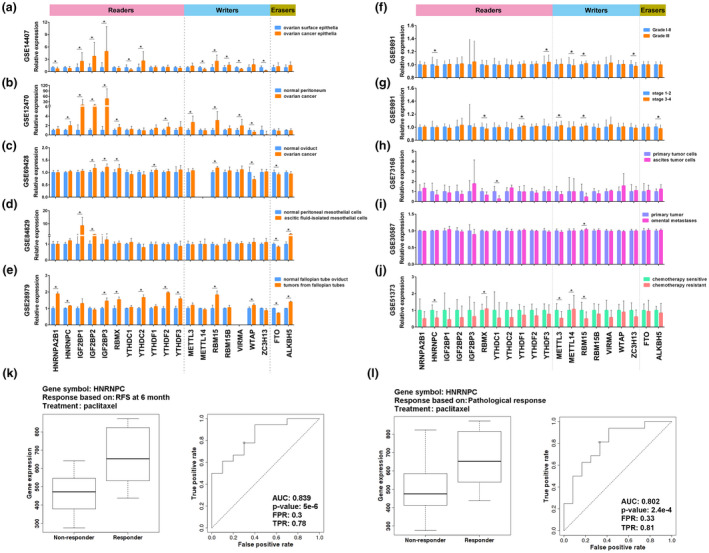
Expression profiles and clinical relevance of m^6^A regulators in ovarian cancer. (a–j) Analysis of differential gene expression of m^6^A regulators between ovarian cancer and ovarian surface epithelial cells in GSE14407 (a), between ovarian cancer and normal peritoneum samples in GSE12470 (b), between HGSOC and paired normal oviduct samples in GSE69428 (c), between ascitic fluid‐isolated mesothelial cells and normal peritoneal mesothelial cells in GSE84829 (d), between early tumors from fallopian tubes of Dicer/PTEN knockout mice and normal mouse fallopian tube oviduct in GSE28979 (e), between different grades in GSE9891 (f), between different stages in GSE9891 (g), between primary ovarian cancer cells and ascites tumor cells in GSE73168 (h), between primary ovarian tumors and metastases from the omentum in GSE30587 (i), and between chemotherapy‐resistant and ‐sensitive samples in GSE51373 (j). (k and l) ROC curves and box‐plots of HNRNPC validated for paclitaxel resistance based on RFS at 6 months (k) and pathological response (l). Error bar ± SD, *p < 0.05

Two GEO databases demonstrated that 7 readers (HNRNPC, IGF2BP1, IGF2BP2, IGF2BP3, RBMX, YTHDC2, and YTHDF2) and 3 writers (METTL3, RBM15, and RBM15B) were more highly expressed in ovarian cancer than in ovarian surface epithelium or normal peritoneum tissues (GEO14407 and GEO12470, Figure 2a,b). Besides that, HNRNPA2B1, YTHDC1, METTL14, WTAP, and ZC3H13 were downregulated in cancer tissues, whereas VIRMA had opposite alterations between these two databases (Figure 2a,b). Given the theory implicating the distal oviduct as a common source for epithelial ovarian cancer, we analyzed the GSE69428 data and showed that the expression of IGF2BP2, IGF2BP3, RBMX, YTDHF1, and RBM15 was also higher in ovarian cancer than in normal oviduct (Figure 2c). Moreover, IGF2BP1, IGF2BP2, and ALKBH5 were upregulated in ascitic fluid isolated mesothelial cells than in normal peritoneal mesothelial cell (GSE84829, Figure 2d). In addition, tumors from fallopian tubes of Dicer/PTEN knockout mice revealed alterations in expression of 7 readers (HNRNPA2B1, HNRNPC, IGF2BP3, RBMX, YTHDC2, YTHDF2, and YTHDF3), 2 writers (RBM15 and WTAP) and 2 erasers (FTO and ALKBH5) in comparison with normal mouse fallopian tube oviduct (GSE28979, Figure 2e).

To determine the clinical relevance of m^6^A regulators in ovarian cancer, we analyzed the relationship between expression alteration of m^6^A regulators and ovarian cancer clinicopathological features. As the pathological grade increased, the expression of YTHDF3 enhanced, while HNRNPC and ZC3H13 decreased (GSE9891, Figure 2f; see also File S1). The significant correlation between pathological stages and expression levels of YTHDF1 and RBM15 was confirmed in GSE9891 data (Figure 2g; see also Table S1). We also noticed that decreased expression of YTHDC1 and increased expression of RBM15 were correlated with the status of ovarian cancer cell metastasis (GSE73168 and GSE30587, Figure 2h,i). Moreover, as the combination of platin plus paclitaxel is the standard first‐line chemotherapy for patients with ovarian cancer, we analyzed the relationship between expression pattern of m^6^A regulators and chemotherapy sensitivity. We found that HNRNPC, METTL3, and RBM15 were downregulated in chemotherapy‐resistant group, while RMBX and METTL14 were increased (Figure 2j). Importantly, the ROC curve showed that increased expression of HNRNPC could perfectly predict response to paclitaxel for ovarian cancer patients based on relapse‐free survival (RFS) at 6 months (AUC =0.839, *p* = 5.0e‐6, Figure 2k and Table 1), RFS at 12 months (AUC =0.802, *p* = 2.4e‐4, see also Table S2) and pathological response (AUC =0.803, *p* = 2.4e‐4, Figure 2l), while decreased expression of YTHDC1 could predict response to paclitaxel on RFS at 6 months (AUC =0.707, *p* = 1.5e‐3, Table 1). These data indicated that m^6^A regulators may play critical roles in ovarian tumorigenesis and function as a predictor of metastasis and chemoresponsiveness.

**Table 1:** Predictive value of m^6^A regulators in response to chemotherapy in ovarian cancer based on relapse‐free survival at 6 months

	Paclitaxel			Platin		
	AUC	ROC *p*‐value	Mann–Whitney test *p*‐value	AUC	ROC *p*‐value	Mann–Whitney test *p*‐value
HNRNPA2B1	0.556	0.31	0.65	0.535	0.17	0.32
HNRNPC	0.839	5.0e‐6	0.0037	0.502	0.48	0.95
IGF2BP1	0.619	0.15	0.31	0.539	0.13	0.27
IGF2BP2	0.578	0.13	0.25	0.508	0.39	0.79
IGF2BP3	0.565	0.19	0.34	0.546	0.062	0.11
RBMX	0.61	0.052	0.11	0.548	4.2e‐02	0.094
YTHDC1	0.707	1.5e‐03	0.0024	0.636	5.8e‐06	1.6e‐06
YTHDC2	0.634	3.0e‐02	0.049	0.625	4.5e‐06	1.1e‐05
YTHDF1	0.571	0.16	0.3	0.599	2.5e‐04	4.8e‐04
YTHDF2	0.566	0.15	0.33	0.657	3.6e‐09	3.2e‐08
YTHDF3	0.66	1.3e‐02	0.019	0.579	3.9e‐03	0.0057
METTL3	0.648	2.1e‐02	0.03	0.669	3.1e‐08	1.4e‐06
METTL14	0.544	0.36	0.72	0.502	0.47	0.94
RBM15	0.517	0.41	0.8	0.575	3.6e‐03	0.0085
RBM15B	0.663	1.5e‐03	0.017	0.577	2e‐03	0.0071
VIRMA	0.522	0.43	0.87	0.527	0.24	0.45
WTAP	0.543	0.27	0.52	0.569	5.4e‐03	0.015
ZC3H13	0.511	0.43	0.87	0.545	4.9e‐02	0.11
FTO	0.606	3.8e‐02	0.12	0.563	1.2e‐02	0.027
ALKBH5	0.636	0.12	0.24	0.502	0.48	0.97

### 3.2 Prognostic value of m^6^A regulators in ovarian cancer

**FIGURE 3:**
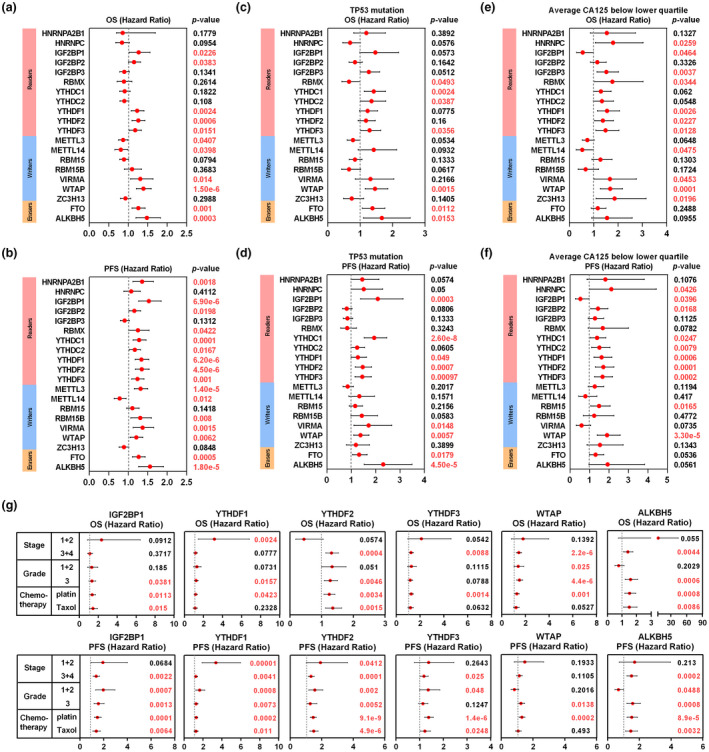
Prognostic value of m^6^A regulators in ovarian cancer. (a and b) The distribution of hazard ratios of OS and PFS across m^6^A regulators in ovarian cancer patients from Kaplan–Meier plotter database (http://kmplot.com/analysis/). (c and d) The distribution of hazard ratios of OS and PFS in patients with TP53 mutation. (e and f) The distribution of hazard ratios of OS and PFS in patients with the status of CA125 level below lower quartile. (g) The distribution of hazard ratios of OS and PFS in patients with different grades, stages, and chemotherapy

Next, we sought to evaluate the predictive value of m^6^A regulators for prognosis in ovarian cancer. Kaplan‐Meier log‐rank analysis revealed that high expressions of YTHDF1 (*p* = 0.0024, HR = 1.23, 95% CI = 1.08–1.41), YTHDF2 (*p* = 0.0006, HR = 1.26, 95% CI = 1.10–1.43), WTAP (*p* = 1.5e‐6, HR = 1.39, 95% CI = 1.21–1.59), FTO (*p* = 0.001, HR = 1.26, 95% CI = 1.10–1.44), and ALKBH5 (*p* = 0.0003, HR = 1.48, 95% CI = 1.20–1.83) were significantly correlated with poor overall survival (OS) (Figure 3a). Besides these genes, high expressions of HNRNPA2B1 (*p* = 0.0018, HR = 1.36, 95% CI = 1.12–1.65), IGF2BP1 (*p* = 6.9e‐6, HR = 1.53, 95% CI = 1.27–1.85), YTHDC1 (*p* = 0.0001, HR = 1.28, 95% CI = 1.13–1.46), YTHDF3 (*p* = 0.001, HR = 1.24, 95% CI = 1.09–1.41), METTL3 (*p* = 1.4e‐5, HR = 1.32, 95% CI = 1.16–1.5), RBM15B (*p* = 0.008, HR = 1.31, 95% CI = 1.07–1.6), and VIRMA (*p* = 0.0015, HR = 1.37, 95% CI = 1.13–1.66) were correlated with worse progression‐free survival (PFS) (Figure 3b). We also evaluated the prognostic value for each m^6^A regulator in ovarian cancer patients with different clinicopathologic features. Among these m^6^A regulators, YTHDC1, YTHDF3, WTAP, FTO, and ALKBH5 were risk prognostic factors with HR >1 for both OS and PFS in patients with TP53 mutation (Figure 3c,d). HNRNPC, YTHDF1, YTHDF2, YTHDF3, and WTAP were correlated with poor OS and PFS with the status of CA125 level below lower quartile (Figure 3e,f). In addition, several risk factors of m^6^A regulators for OS and PFS in ovarian cancer patients with different grade, stage, and chemotherapy were assessed (Figure 3g). For instance, compared with YTHDF2^low^ group, YTHDF2^high^ group had shorter OS and PFS in patients treated with platin or Taxol, whereas no differences were found in patients with low pathological stage. Besides, high expression of ALKBH5 was a risk factor for OS and PFS in patients with high pathological grade and stage (Figure 3g). These results highlighted potential roles of m^6^A regulators as prognostic markers in ovarian cancer patients.

### 3.3 Oncogenic pathways regulated by m^6^A regulators in ovarian cancer

**FIGURE 4:**
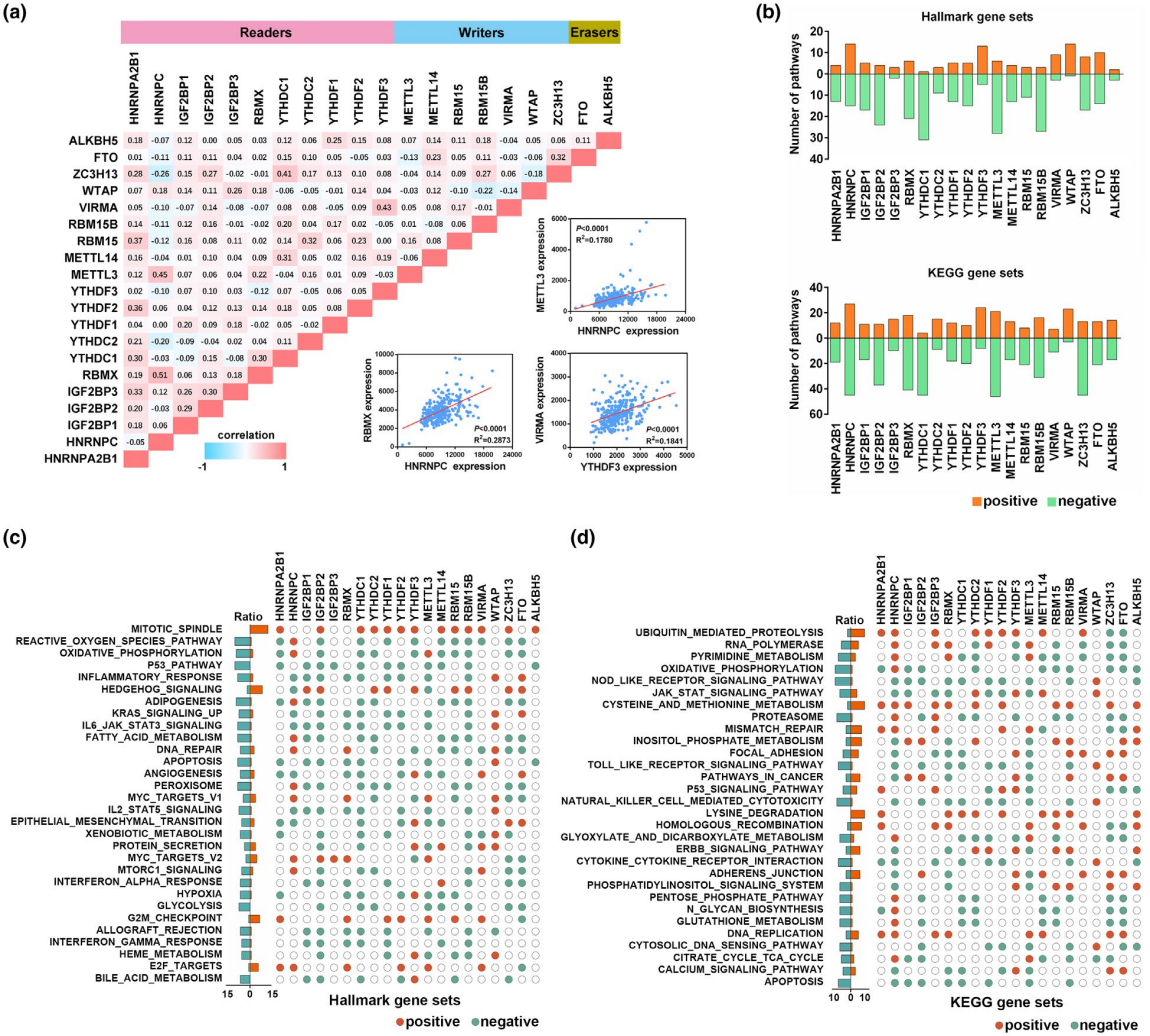
Oncogenic pathways regulated by m^6^A regulators in ovarian cancer. (a) Correlation among the expression of m^6^A regulators. The scatter plot shows the correlation between METTL3 and HNRNPC, RBMX and HNRNPC, and VIRMA and YTHDF3. (b) The number of Hallmark gene sets (upper panel) and KEGG gene sets (lower panel) is correlated with individual m^6^A regulators. (c) The correlation between m^6^A regulators and Hallmark gene sets. (d) The correlation between m^6^A regulators and KEGG gene sets

To better understand the functions of m^6^A regulators in ovarian cancer, we first analyzed the correlation among these regulators. As shown in Figure 4a, the expressions of m^6^A regulators were not only correlated with several regulators in the same functional type but also among different types. For example, the expression of HNRNPA2B1 was positively correlated with the expressions of IGF2BP3, YTHDC1, YTHDF2, RBM15, and ZC3H13 in ovarian cancer. Similarly, the expression of HNRNPC was positively correlated with RBMX and METTL3, and negatively correlated with YTHDC2 and ZC3H13.

Then, we investigated the oncogenic pathways potentially regulated by m^6^A regulators in ovarian cancer. GSEA analysis demonstrated that the expressions of METTL3, YTHDC1, RBM15B, HNRNPC, IGF2BP2, RBMX, and ZC3H13 were correlated with a higher number of multiple Hallmark pathways in ovarian cancer (Figure 4b). Upregulated expressions of m^6^A regulators were enriched in the activation of several cancer‐related pathways, such as mitotic spindle, Hedgehog signaling, MYC targets, G2M checkpoint, and E2F target, whereas reactive oxygen species pathway, oxidative phosphorylation, p53 pathway, inflammatory response, adipogenesis, IL6/JAK/STAT3 signaling, fatty acid metabolism, apoptosis, and peroxisome were negatively correlated with the expression of m^6^A regulators (Figure 4c). We also performed KEGG pathway enrichment to recognize biological processes regulated by m^6^A regulators. Similarly, the expressions of HNRNPC, METTL3, RBMX, ZC3H13, YTHDC1, IGF2BP2, and RBM15B were correlated with a higher number of KEGG pathways (Figure 4b). Our results also indicated that upregulated m^6^A regulators were positively enriched in ubiquitin‐mediated proteolysis, cysteine and methionine metabolism, lysine degradation, and homologous recombination, whereas oxidative phosphorylation, NOD‐like receptor signaling pathway, proteasome, natural killer cell‐mediated cytotoxicity, pyrimidine metabolism, and Toll‐like receptor signaling pathway were negatively correlated with the expression of m^6^A regulators (Figure 4d).

### 3.4 Correlation between immune cell infiltration and the expression of m^6^A regulators in ovarian cancer

**FIGURE 5:**
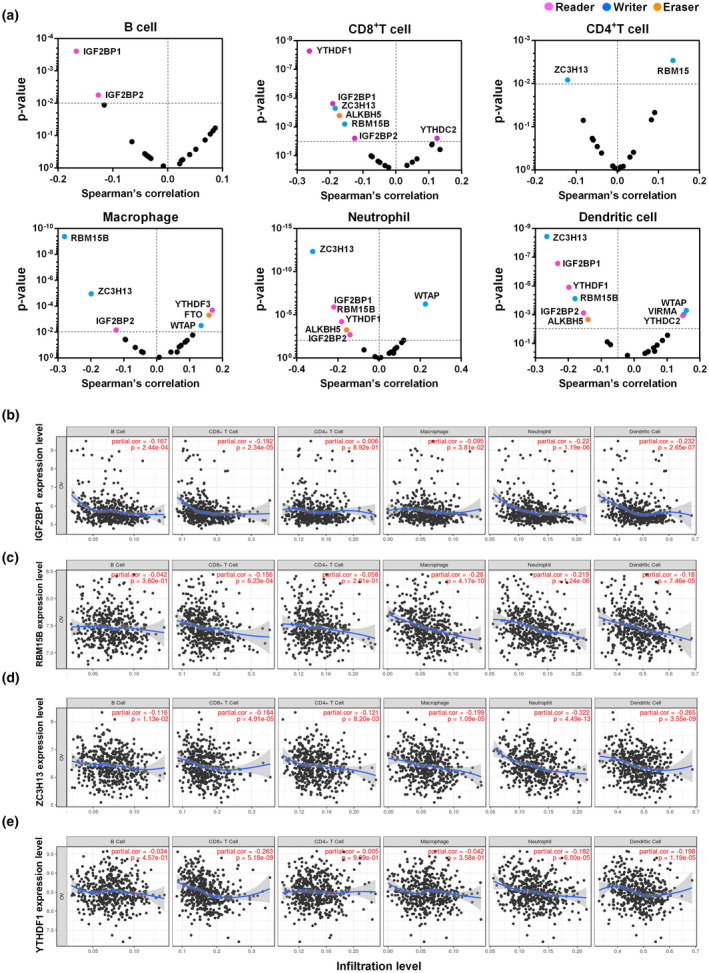
Correlation between immune cell infiltration and the expression of m^6^A regulators in ovarian cancer. (a) Correlation between the expression of m^6^A regulators and infiltrating levels of B cells, CD8^+^ T cells, CD4^+^ T cells, macrophages, neutrophils, and dendritic cells in ovarian cancer. (b–e) Correlation between the expression of specific m^6^A regulators (IGF2BP1, RBM15B, ZC3H13, and YTHDF1) and infiltrating levels of B cells, CD8^+^ T cells, CD4^+^ T cells, macrophages, neutrophils, and dendritic cells in ovarian cancer

Infiltration of lymphocytes is an independent predictor of ovarian cancer patient survival and chemoresistance. Hence, we explored whether the expression of m^6^A regulators was correlated with immune infiltration levels in ovarian cancer. The results showed that several m^6^A regulators, including 4 readers (IGF2BP1, IGF2BP2, YTHDF1, and YTHDC2), 3 writers (ZC3H13, RBM15B, and WTAP), and 1 eraser (ALKBH5) had significant correlations with immune cell infiltration levels (Figure 5a). In particular, IGF2BP1 expression level had significant negative correlation with infiltrating of B cells (Cor = −0.167, *p* = 2.4e‐4), CD8^+^ T cells (Cor = −0.192, *p* = 2.3e‐5), neutrophils (Cor = −0.220, *p* = 1.2e‐6), and dendritic cells (Cor = −0.232, *p* = 2.7e‐7) (Figure 5b). RBM15B expression level had significantly negative correlation with infiltrating of CD8^+^ T cells (Cor = −0.156, *p* = 6.2e‐4), macrophages (Cor = −0.280, *p* = 4.2e‐10), neutrophils (Cor = −0.220, *p* = 1.2e‐6), and dendritic cells (Cor = −0.180, *p* = 7.5e‐5) (Figure 5c). Similarly, the expression of ZC3H13 was significantly negatively correlated with infiltrating level of CD8^+^ T cells (Cor = −0.184, *p* = 4.9e‐5), CD4^+^ T cells (Cor = −0.121, *p* = 8.2e‐3), macrophages (Cor = −0.199, *p* = 1.1e‐5), neutrophils (Cor = −0.322, *p* = 4.5e‐13), and dendritic cells (Cor = −0.265, *p* = 3.6e‐9) (Figure 5d). The expression of YTHDF1 was significantly negatively correlated with infiltrating level of CD8^+^ T cells (Cor = −0.263, *p* = 5.2e‐9), neutrophils (Cor = −0.182, *p* = 6.0e‐5), and dendritic cells (Cor = −0.198, *p* = 1.2e‐5) (Figure 5e). Moreover, similar correlations were also observed across different types of cancers (see Figure S1). These findings propose that the expressions of specific m^6^A regulators may be correlated with immune cell infiltration in ovarian cancer.

**FIGURE 6:**
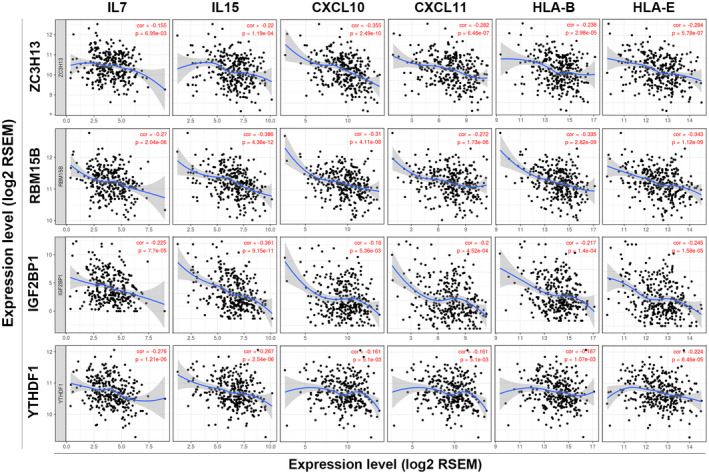
Correlation between the expression of specific m^6^A regulators (IGF2BP1, RBM15B, ZC3H13, and YTHDF1) and immune‐related factors (IL7, IL15, CXCL10, CXCL11, HLA‐B, and HLA‐E)

We further investigated the relationship between these immune infiltration‐related m^6^A regulators and immune marker genes of diverse immune cells, including B cells, T cells (general), CD8^+^ T cells, Th1 cells, Th2 cells, Treg cells, tumor‐associated macrophages (TAM), M1 and M2 macrophages, neutrophils, natural killer (NK) cells, and dendritic cells in ovarian cancer. As shown in Table 2, the expression of RBM15B was significantly correlated with most immune marker genes of different T cells and various immune cells. ZC3H13 expression had correlation with immune marker genes of CD8+ T cells, TAM, M2 macrophages, and dendritic cells. The expression of YTHDF1 was correlated with immune marker genes of T cells (general), CD8^+^ T cells, and dendritic cells, whereas IGF2BP1 expression had correlation only with dendritic cells. Our results also showed significant correlations between two immune infiltration‐related m^6^A regulators (RBM15B and ZC3H13) and marker genes of T‐cell exhaustion, including PDCD1 (PD‐1), CD274 (PD‐L1), CTLA4, LAG3, and GZMB (Table 2). Moreover, we found that the expression of these immune infiltration‐related m^6^A regulators was also significantly correlated with several interleukins (IL1B, IL7, IL15, and IL18), CC and CXC chemokines (CCL2, CCL5, CXCL10, CXCL11, and CXCL17), and human leukocyte antigens (HLA‐A, HLA‐B, HLA‐C, HLA‐E, and HLA‐F) (Figure 6; see also Table S3). Therefore, these data confirmed the findings that the expressions of specific m^6^A regulators were associated with tumor immune cell infiltration.

**Table 2:** Correlation analysis between m^6^A regulators and related gene markers of immune cells

Immune cells	Gene markers	ZC3H13		RBM15B		IGF2BP1		YTHDF1	
		Cor	*p*	Cor	*p*	Cor	*p*	Cor	*p*
B cell	CD19	−0.02	0.73	−0.02	0.69	0.19	**	0.10	0.08
	CD79A	−0.03	0.56	−0.13	0.02	0.02	0.67	0.03	0.63
T cell (general)	CD2	−0.16	*	−0.31	***	−0.14	0.14	−0.20	**
	CD3D	−0.18	*	−0.31	***	−0.13	0.02	−0.22	**
	CD3E	−0.12	0.03	−0.29	***	−0.14	0.01	−0.21	**
CD8+ T cell	CD8A	−0.16	*	−0.32	***	−0.10	0.08	−0.15	*
	CD8B	−0.16	*	−0.27	***	0.05	0.37	−0.03	0.64
Th1	IFN‐γ	−0.15	0.01	−0.30	***	−0.09	0.13	−0.15	*
	TBX21	−0.11	0.04	−0.24	***	−0.12	0.03	−0.19	*
	TNF‐α	−0.06	0.28	−0.14	0.02	0.02	0.77	−0.16	0.04
Th2	GATA3	0.07	0.22	−0.13	0.03	0.03	0.65	−0.09	0.12
	STAT6	0.05	0.38	0.00	0.96	−0.15	0.01	−0.14	0.01
	IL13	0.02	0.70	−0.05	0.41	−0.05	0.37	−0.12	0.04
Treg	CCR8	−0.09	0.13	−0.14	0.02	0.01	0.83	−0.11	0.06
	FOXP3	−0.09	0.13	−0.21	**	0.00	0.96	−0.06	0.28
	STAT5B	0.34	***	0.20	**	0.08	0.16	0.14	0.15
TAM	CD68	−0.25	***	−0.32	***	−0.09	0.14	−0.15	*
	CCL2	−0.21	**	−0.25	***	−0.15	0.01	−0.23	***
	IL10	−0.15	0.01	−0.24	***	0.00	0.91	−0.10	0.08
M1 macrophage	NOS2	−0.02	0.75	0.00	0.99	0.19	*	0.10	0.10
	PTGS2	0.04	0.52	−0.02	0.72	0.03	0.63	−0.09	0.13
	IRF5	−0.15	0.01	−0.22	**	−0.03	0.66	−0.06	0.28
M2 macrophage	CD163	−0.12	0.04	−0.23	***	−0.02	0.79	−0.08	0.16
	MS4A4A	−0.21	**	−0.33	***	−0.07	0.26	−0.15	0.01
	VSIG4	−0.25	***	−0.31	***	−0.06	0.28	−0.09	0.12
Neutrophils	CCR7	−0.07	0.24	−0.19	**	−0.05	0.36	−0.20	**
	CEACAM8	0.27	***	0.00	0.89	0.07	0.23	0.02	0.76
	ITGAM	−0.11	0.05	−0.20	**	−0.07	0.23	−0.14	0.13
NK cell	KIR2DL1	0.03	0.64	−0.10	0.08	−0.01	0.91	−0.09	0.13
	KIR2DL3	−0.06	0.28	−0.18	*	−0.21	**	−0.13	0.03
	KIR3DL1	0.00	0.97	−0.15	*	−0.12	0.04	−0.07	0.20
	KIR3DL2	−0.02	0.74	−0.15	0.01	−0.10	0.10	−0.13	0.02
Dendritic cell	HLA‐DPB1	−0.31	***	−0.26	***	−0.30	***	−0.26	***
	HLA‐DQB1	−0.175	*	−0.21	**	−0.23	***	−0.19	**
	HLA‐DRA	−0.36	***	−0.32	***	−0.31	***	−0.27	***
	ITGAX	−0.08	0.16	−0.22	**	−0.11	0.05	−0.18	*
T‐cell exhaustion	PDCD1	−0.16	*	−0.28	***	−0.04	0.53	−0.12	0.04
	CD274	−0.17	*	−0.25	***	−0.14	0.01	−0.21	**
	CTLA4	−0.22	**	−0.29	***	−0.07	0.20	−0.10	0.08
	LAG3	−0.22	**	−0.32	***	−0.06	0.28	−0.12	0.04
	GZMB	−0.20	**	−0.33	***	−0.14	0.01	−0.19	*

Abbreviation:TAM, tumor‐associated macrophage; Th, T helper cells; Treg, regulatory T cells; NK, natural killer; Cor, R value of Spearman’s correlation

^*^(*p* < 0.01).

^**^(*p* < 0.001).

^***^(*p* < 0.0001).

## 4 DISCUSSION

Herein, we demonstrated that changes in m^6^A regulator expression were associated with malignancy and prognosis of ovarian cancer. Increased expression of YTHDF3, WTAP, FTO, and ALKBH5 was associated with shorter OS and PFS regardless of the status of TP53 mutation. We found that a decrease in YTHDC1 and an increase in RBM15 expressions were correlated with ovarian cancer cell metastases. We also suggested that HNRNPC was a predictor of paclitaxel resistance. In addition, GSEA analysis showed that the mechanism of m^6^A regulators regulating ovarian cancer was related to a variety of tumor‐related pathways. Importantly, our data showed that immune cell infiltration levels and various immune gene markers were closely associated with the expression of m^6^A regulators, suggesting that RBM15B, ZC3H13, YTHDF1, and IGF2BP1 might play the role of immune infiltration‐related m^6^A regulators in ovarian cancer. Thus, our current study provided insights into the value of m^6^A regulators in the determination of prognosis and understanding of their potential roles in ovarian cancer immunology.

RNA m^6^A methylation is a widespread modification that regulates selective control of gene expression (Dominissini et al., 2012; Li, Tong, et al., 2017; Yue et al., 2015). Research on the roles of m^6^A readers, writers, and erasers have improved our understanding of physiological and pathological significance of RNA methylation (Meyer & Jaffrey, 2017). Accumulating evidences suggest that these m^6^A methylation regulators function as oncogenes or tumor‐suppressor genes and are involved in the proliferation, differentiation, invasion, and metastasis of cancer cells (Lan et al., 2019). Recent years, the clinical relevance and molecular characteristics of m^6^A regulators in different cancer types have been reported (Chai et al., 2019; Kwok et al., 2017; Li et al., 2019; Su et al., 2019; Zhou et al., 2019). Although some studies have demonstrated that the m^6^A writer METTL3, reader IGF2BP1, and eraser ALKBH5 are involved in the development of ovarian cancer (Hua et al., 2018; Müller et al., 2019; Zhu et al., 2019), little is known about the role of other m^6^A regulators in ovarian cancer.

Here, we comprehensively evaluated the expression alterations of 20 m^6^A regulators in different databases. Compared with normal ovarian surface epithelium, peritoneum, and oviduct tissues, increased or decreased expression of several specific m^6^A regulators were found in ovarian cancer tissues and ascitic fluid‐isolated cells. The relationship between the expression of m^6^A regulators and clinicopathological characteristics, such as grading, staging, metastasis,, and chemotherapy response was also confirmed in our study. Moreover, HNRNPC, a member of ubiquitously expressed heterogeneous nuclear ribonucleoproteins (hnRNPs) family, which influences pre‐mRNA processing and mRNA transport and metabolism (Fischl et al., 2019), was downregulated in chemotherapy‐resistant group and upregulated in paclitaxel response group. The ROC/AUC score was also high, indicating its predictive value of paclitaxel response in ovarian cancer. Our study also revealed that m^6^A regulators might be correlated with several tumor‐related signaling pathways and biological processes in ovarian cancer, including Hedgehog signaling, p53 pathway, Myc‐dependent pathway, reactive oxygen species pathway, IL6/JAK/STAT3 signaling, apoptosis, mitotic spindle, proteolysis, amino acid metabolism, homologous recombination, etc.

Previous studies have shown that alterations in m^6^A regulators are associated with poor patient outcome (Chai et al., 2019; Kwok et al., 2017; Li et al., 2019; Su et al., 2019; Zhou et al., 2019). In our current study, according to the Kaplan–Meier plotter database, when YTHDF1, YTHDF2, WTAP, FTP, and ALKBH5 were highly expressed in ovarian cancer, they were validated as valuable prognostic risk factors for low OS and PFS with high HR. This observation supports our hypothesis that specific m^6^A regulators are promising candidate biomarkers for predicting the prognosis of patients with ovarian cancer. Moreover, we also established some m^6^A regulators for the prognostic value of ovarian cancer with the status of TP53 mutation, CA125 level, different grades/stages, and chemotherapy. Additionally, our analysis showed opposing correlations between members with similar functional directionality and ovarian cancer patient outcomes, indicating the functional diversity of m^6^A regulators.

Ovarian cancer microenvironment plays a critical role in controlling the cancer cell fate, treatment, and prognosis (Yin et al., 2019). In recent studies, a new concept of immune regulatory function of m^6^A regulatory factor has been proposed. Han et al. (2019) reported that the loss of the reader YTHDF1 in dendritic cells restricted the expression of lysosomal proteases, promoted cross‐presentation of tumor antigens, improved cross‐priming of CD8^+^ T cells, and enhanced therapeutic efficacy of PD‐L1 blockade. The writer METTL3 has been revealed to catalyze m^6^A of membrane co‐stimulatory molecules CD40, CD80, and TLR signaling adaptor TIRAP during dendritic cells maturation, and enhanced their translation for promoting T‐cell activation (Wang et al., 2019). Besides that, METTL3 and YTHDF2 also served as negative regulators of type I interferon response to control the innate immune response (Winkler et al., 2019). Hence, the important aspect of our study is to emphasize the role of m^6^A regulators in immune cell infiltration and immune escape in ovarian cancer. We demonstrated four immune infiltration‐related m^6^A regulators in ovarian cancer, including RBM15B, ZC3H13, YTHDF1, and IGF2BP1. Specifically, (1) GSEA analyses revealed that inflammatory response, interferon response, NOD‐like receptor, and Toll‐like receptor pathways were negatively correlated with high expression of these m^6^A regulators. (2) There was a strong association of the expression level of these m^6^A regulators with the infiltration level of immune cells (B cells, CD8^+^ T cells, CD4^+^ T cells, macrophages, neutrophils, and dendritic cells). (3) The expression level of these m^6^A regulators had a strong correlation with diverse immune marker genes, interleukins, CC and CXC chemokines, and human leukocyte antigens. (4) The increased expression of these m^6^A regulators correlates with the expression of T‐cell exhaustion markers (PD‐1, CD274, CTLA4, LAG3, and GZMB). Therefore, the cross‐talk between the expressions of m^6^A regulators and tumor microenvironment might be an important mechanism for the development and progression of ovarian cancer. Nevertheless, more functional and mechanism experiments are needed for further verification.

In summary, our results systematically demonstrated expression alterations and prognostic value of m^6^A regulators in ovarian cancer. The expressions of several specific m^6^A regulators were correlated with cancer‐related pathways, tumor metastasis, and chemotherapy resistance. In addition, the expressions of m^6^A regulators might be involved in the regulation of immune cell infiltration and immune escape. Therefore, our study provides new insights into the role of m^6^A regulators in ovarian cancer.

## CONFLICT OF INTEREST

The authors declare that they have no conflicts of interest.

## AUTHOR CONTRIBUTIONS

JWZ and QYW contributed to the study conception and design. QYW, QYZ, QXL, and JZ collected and analyzed the data. The first draft of the manuscript was written by QYW, QYZ and JWZ. All the authors read and approved the final manuscript.

## Supporting information

Fig S1

Table S1

Table S2

Table S3
