# Supplementary figures and images for: Clinicopathological and immunological characterization of RNA m6A methylation regulators in ovarian cancer
Source: Mol Genet Genomic Med. 2020 Nov 22;9(1):e1547. doi: 10.1002/mgg3.1547 (PMC7963423; doi:10.1002/mgg3.1547)

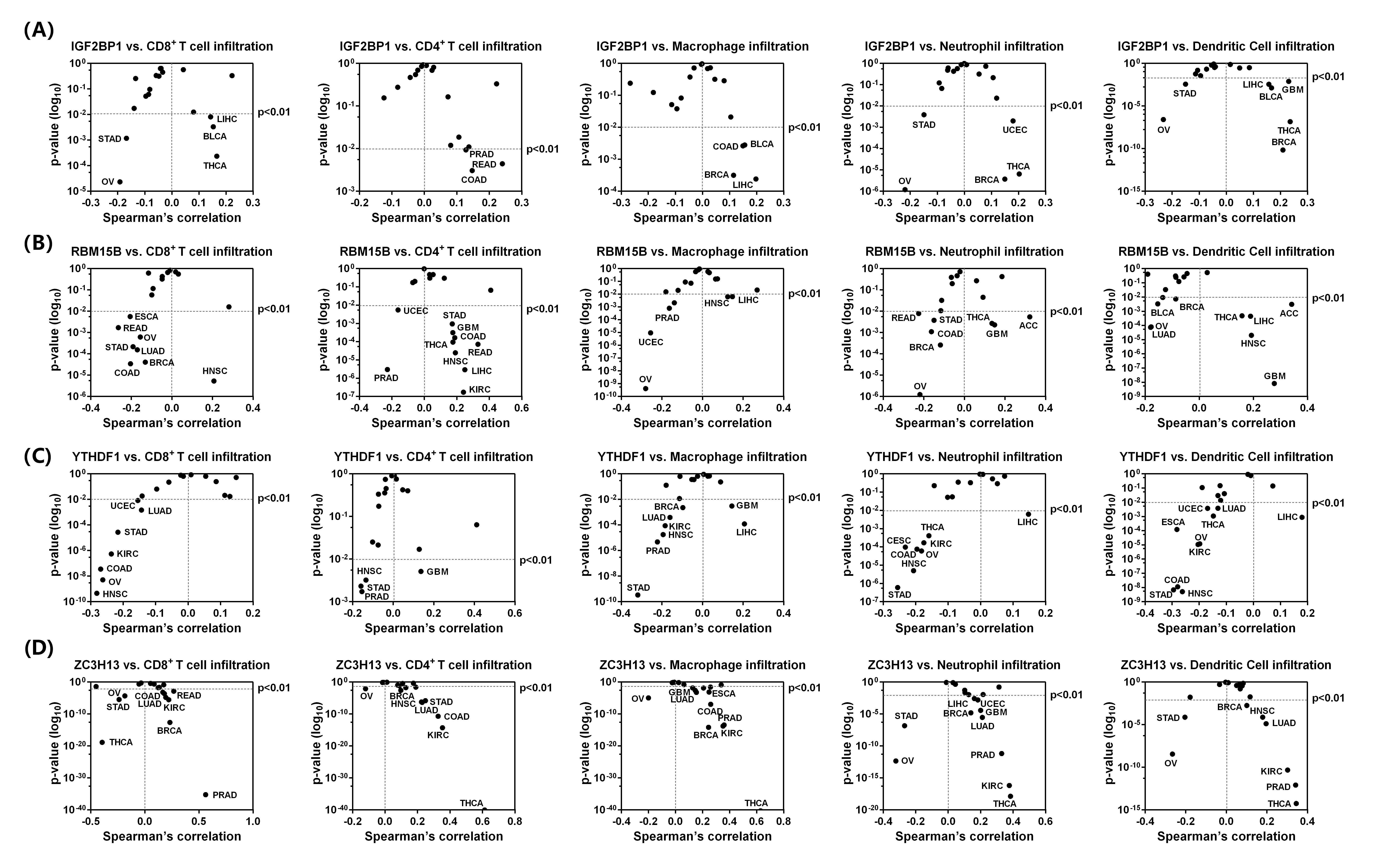

Supplement: Supplementary file 1 — Fig S1 [file MGG3-9-e1547-s001.tif]
