# Supplementary material for: Clinicopathological and immunological characterization of RNA m6A methylation regulators in ovarian cancer
Source: Mol Genet Genomic Med. 2020 Nov 22;9(1):e1547. doi: 10.1002/mgg3.1547 (PMC7963423; doi:10.1002/mgg3.1547)
Supplement: Supplementary file 2 — Table S1 [file MGG3-9-e1547-s003.docx]

**Supplementary Table S1**. Association between the expression of m^6^A regulators and pathologic grade/stage in GES9891.

|  |  | Grade (n=280) | | | | Stage (n=281) | | | |
| --- | --- | --- | --- | --- | --- | --- | --- | --- | --- |
|  |  | I-II | III | χ^2^ | P-value | 1-2 | 3-4 | χ^2^ | P-value |
| HNRNPA2B1 | low | 53 | 87 | 2.470 | 0.1160 | 25 | 115 | 1.859 | 0.1727 |
|  | high | 66 | 74 |  |  | 17 | 124 |  |  |
| HNRNPC | low | 45 | 95 | 12.29 | 0.0005 | 17 | 123 | 1.725 | 0.1890 |
|  | high | 74 | 66 |  |  | 25 | 116 |  |  |
| IGF2BP1 | low | 65 | 75 | 1.768 | 0.1836 | 13 | 127 | 7.033 | 0.0080 |
|  | high | 54 | 86 |  |  | 29 | 112 |  |  |
| IGF2BP2 | low | 63 | 77 | 0.716 | 0.3974 | 24 | 116 | 1.059 | 0.3035 |
|  | high | 56 | 84 |  |  | 18 | 123 |  |  |
| IGF2BP3 | low | 68 | 72 | 4.224 | 0.0399 | 18 | 122 | 0.958 | 0.3276 |
|  | high | 51 | 89 |  |  | 24 | 117 |  |  |
| RBMX | low | 55 | 85 | 1.184 | 0.2766 | 16 | 124 | 2.716 | 0.0993 |
|  | high | 64 | 76 |  |  | 26 | 115 |  |  |
| YTHDC1 | low | 60 | 80 | 0.015 | 0.9038 | 18 | 122 | 0.958 | 0.3276 |
|  | high | 59 | 81 |  |  | 24 | 117 |  |  |
| YTHDC2 | low | 58 | 82 | 0.132 | 0.7169 | 17 | 123 | 1.725 | 0.189 |
|  | high | 61 | 79 |  |  | 25 | 116 |  |  |
| YTHDF1 | low | 59 | 81 | 0.015 | 0.9038 | 30 | 110 | 9.221 | 0.0024 |
|  | high | 60 | 80 |  |  | 12 | 129 |  |  |
| YTHDF2 | low | 63 | 77 | 0.716 | 0.3974 | 25 | 115 | 1.859 | 0.1727 |
|  | high | 56 | 84 |  |  | 17 | 124 |  |  |
| YTHDF3 | low | 72 | 68 | 9.134 | 0.0025 | 24 | 116 | 1.059 | 0.3035 |
|  | high | 47 | 93 |  |  | 18 | 123 |  |  |
| METTL3 | low | 58 | 82 | 0.132 | 0.7169 | 26 | 114 | 2.884 | 0.0895 |
|  | high | 61 | 79 |  |  | 16 | 125 |  |  |
| METTL14 | low | 54 | 86 | 1.768 | 0.1836 | 23 | 117 | 0.482 | 0.4875 |
|  | high | 65 | 75 |  |  | 19 | 122 |  |  |
| RBM15 | low | 67 | 73 | 3.288 | 0.0698 | 30 | 110 | 9.221 | 0.0024 |
|  | high | 52 | 88 |  |  | 12 | 129 |  |  |
| RBM15B | low | 63 | 77 | 0.716 | 0.3974 | 20 | 119 | 0.067 | 0.7952 |
|  | high | 56 | 84 |  |  | 22 | 120 |  |  |
| VIRMA | low | 64 | 76 | 1.184 | 0.2766 | 27 | 113 | 4.132 | 0.0421 |
|  | high | 55 | 85 |  |  | 15 | 126 |  |  |
| WTAP | low | 58 | 82 | 0.132 | 0.7169 | 25 | 115 | 1.859 | 0.1721 |
|  | high | 61 | 79 |  |  | 17 | 124 |  |  |
| ZC3H13 | low | 45 | 95 | 12.29 | 0.0005 | 17 | 123 | 1.725 | 0.189 |
|  | high | 74 | 66 |  |  | 25 | 116 |  |  |
| FTO | low | 56 | 84 | 0.716 | 0.3974 | 18 | 122 | 0.958 | 0.3276 |
|  | high | 63 | 77 |  |  | 24 | 117 |  |  |
| ALKBH5 | low | 63 | 77 | 0.716 | 0.3974 | 17 | 123 | 1.725 | 0.189 |
|  | high | 56 | 84 |  |  | 25 | 116 |  |  |
