# Supplementary material for: Clinicopathological and immunological characterization of RNA m6A methylation regulators in ovarian cancer
Source: Mol Genet Genomic Med. 2020 Nov 22;9(1):e1547. doi: 10.1002/mgg3.1547 (PMC7963423; doi:10.1002/mgg3.1547)
Supplement: Supplementary file 3 — Table S2 [file MGG3-9-e1547-s002.docx]

**Supplementary Table S2**. Predictive value of m^6^A regulators in response to chemotherapy in ovarian cancer based on relapse-free survival at 12 months.

|  | Paclitaxel | | | Platin | | |
| --- | --- | --- | --- | --- | --- | --- |
|  | AUC | ROC  p-value | Mann-Whitney test  p-value | AUC | ROC  p-value | Mann-Whitney test  p-value |
| HNRNPA2B1 | 0.526 | 0.41 | 0.84 | 0.507 | 0.39 | 0.78 |
| HNRNPC | 0.802 | 2.4e-04 | 0.0076 | 0.511 | 0.35 | 0.69 |
| IGF2BP1 | 0.661 | 0.067 | 0.16 | 0.507 | 0.4 | 0.8 |
| IGF2BP2 | 0.514 | 0.38 | 0.76 | 0.507 | 0.35 | 0.7 |
| IGF2BP3 | 0.517 | 0.36 | 0.71 | 0.518 | 0.17 | 0.34 |
| RBMX | 0.525 | 0.3 | 0.59 | 0.529 | 0.056 | 0.12 |
| YTHDC1 | 0.651 | 3.8e-04 | 0.0012 | 0.553 | 2.4e-03 | 0.0044 |
| YTHDC2 | 0.542 | 0.19 | 0.37 | 0.579 | 5.8e-06 | 1.8e-05 |
| YTHDF1 | 0.603 | 1.2e-02 | 0.027 | 0.558 | 8.2e-04 | 0.0017 |
| YTHDF2 | 0.546 | 0.17 | 0.32 | 0.57 | 8.6e-05 | 1.5e-04 |
| YTHDF3 | 0.524 | 0.31 | 0.6 | 0.534 | 3.2e-02 | 0.061 |
| METTL3 | 0.57 | 0.061 | 0.14 | 0.587 | 5.3e-04 | 0.0011 |
| METTL14 | 0.568 | 0.28 | 0.57 | 0.512 | 0.32 | 0.64 |
| RBM15 | 0.542 | 0.19 | 0.37 | 0.525 | 0.084 | 0.17 |
| RBM15B | 0.57 | 0.066 | 0.13 | 0.516 | 0.2 | 0.4 |
| VIRMA | 0.505 | 0.48 | 0.98 | 0.505 | 0.42 | 0.85 |
| WTAP | 0.6 | 1.7e-02 | 0.032 | 0.58 | 4.5e-06 | 1.4e-05 |
| ZC3H13 | 0.559 | 0.1 | 0.21 | 0.512 | 0.25 | 0.51 |
| FTO | 0.567 | 0.071 | 0.15 | 0.568 | 1e-04 | 2.5e-04 |
| ALKBH5 | 0.68 | 4.6e-02 | 0.11 | 0.533 | 0.11 | 0.22 |
