# Supplementary material for: Clinicopathological and immunological characterization of RNA m6A methylation regulators in ovarian cancer
Source: Mol Genet Genomic Med. 2020 Nov 22;9(1):e1547. doi: 10.1002/mgg3.1547 (PMC7963423; doi:10.1002/mgg3.1547)
Supplement: Supplementary file 4 — Table S3 [file MGG3-9-e1547-s004.docx]

**Supplementary Table S3**. Correlation analysis between m^6^A regulators and immunity factors.

| Gene markers | ZC3H13 | | RBM15B | | IGF2BP1 | | YTHDF1 | |
| --- | --- | --- | --- | --- | --- | --- | --- | --- |
|  | Cor | P | Cor | P | Cor | P | Cor | P |
| IL1B | -0.21 | ** | -0.21 | ** | -0.13 | 0.02 | -0.14 | 0.02 |
| IL4 | 0.06 | 0.3 | 0.04 | 0.48 | -0.03 | 0.65 | -0.11 | 0.07 |
| IL6 | -0.09 | 0.11 | -0.23 | *** | 0.01 | 0.92 | -0.07 | 0.26 |
| IL7 | -0.16 | * | -0.27 | *** | -0.23 | *** | -0.28 | *** |
| IL12A | -0.13 | 0.02 | -0.25 | *** | -0.08 | 0.18 | -0.01 | 0.85 |
| IL12B | 0.00 | 0.93 | -0.22 | ** | -0.07 | 0.23 | -0.10 | 0.11 |
| IL15 | -0.22 | ** | -0.39 | *** | -0.36 | *** | -0.27 | *** |
| IL18 | -0.17 | * | -0.29 | *** | -0.28 | *** | -0.29 | *** |
| IL18RAP | -0.11 | 0.07 | -0.313 | *** | -0.19 | 0.02 | -0.18 | * |
| IL21 | -0.05 | 0.36 | -0.17 | * | -0.05 | 0.43 | 0.05 | 0.35 |
| CXCL1 | -0.12 | 0.04 | -0.16 | * | -0.11 | 0.05 | -0.16 | * |
| CXCL2 | -0.10 | 0.07 | -0.16 | * | -0.14 | 0.02 | -0.20 | ** |
| CXCL10 | -0.36 | *** | -0.31 | *** | -0.16 | * | -0.16 | * |
| CXCL11 | -0.28 | *** | -0.27 | *** | -0.2 | ** | -0.17 | * |
| CXCL12 | 0.05 | 0.40 | -0.10 | 0.08 | -0.11 | 0.06 | -0.04 | 0.51 |
| CXCL17 | -0.22 | ** | -0.21 | ** | -0.37 | *** | -0.16 | * |
| CCL2 | -0.21 | ** | -0.25 | *** | -0.15 | 0.01 | -0.23 | *** |
| CCL3 | -0.18 | * | -0.27 | *** | -0.14 | 0.01 | -0.09 | 0.11 |
| CCL5 | -0.19 | * | -0.33 | *** | -0.15 | * | -0.23 | *** |
| CCL20 | -0.19 | ** | -0.13 | 0.02 | -0.09 | 0.14 | -0.22 | ** |
| HLA-A | -0.24 | *** | -0.31 | *** | -0.17 | * | -0.14 | 0.02 |
| HLA-B | -0.24 | *** | -0.34 | *** | -0.22 | ** | -0.19 | * |
| HLA-C | -0.23 | *** | -0.34 | *** | -0.22 | ** | -0.14 | 0.01 |
| HLA-E | -0.28 | *** | -0.34 | *** | -0.25 | *** | -0.22 | *** |
| HLA-F | -0.26 | *** | -0.34 | *** | -0.25 | *** | -0.19 | ** |

Cor, R value of Spearman’s correlation; *P < 0.01; **P < 0.001; ***P < 0.0001.
